# Cancer testis antigens and genomic instability: More than immunology^☆,☆☆^

**DOI:** 10.1016/j.dnarep.2021.103214

**Published:** 2021-08-17

**Authors:** Ash Jay, Diedre Reitz, Satoshi H. Namekawa, Wolf-Dietrich Heyer

**Affiliations:** a Department of Microbiology and Molecular Genetics, University of California, Davis, Davis, CA, 95616-8665, USA; b Department of Molecular and Cellular Biology, University of California, Davis, Davis, CA, 95616-8665, USA

**Keywords:** BRCAness, Homologous recombination, Meiosis, Poly (ADP-ribose) polymerase inhibition, Replication fork protection

## Abstract

Cancer testis antigens or genes (CTA, CTG) are predominantly expressed in adult testes while silenced in most or all somatic tissues with sporadic expression in many human cancers. Concerted misexpression of numerous CTA/CTGs is rarely observed. This finding argues against the germ cell theory of cancer. A surprising number of CTA/CTGs are involved in meiotic chromosome metabolism and specifically in meiotic recombination. Recent discoveries with a group of CTGs established that their misexpression in somatic cells results in genomic instability by interfering with homologous recombination (HR), a DNA repair pathway for complex DNA damage such as DNA double-stranded breaks, interstrand crosslinks, and single-stranded DNA gaps. HR-deficient tumors have specific vulnerabilities and show synthetic lethality with inhibition of polyADP-ribose polymerase, opening the possibility that expression of CTA/CTGs that result in an HR-defect could be used as an additional biomarker for HR status. Here, we review the repertoire of CTA/CTGs focusing on a cohort that functions in meiotic chromosome metabolism by interrogating relevant cancer databases and discussing recent discoveries.

## 1. Introduction

Cancer testis antigens (**CTA**) describe a group of proteins that are predominantly expressed in adult testes while silenced in most other somatic tissues [1,2]. For many CTAs, their antigen status has not been established, and they are referred to in this review as cancer testis genes (**CTG**). CTA/CTGs are frequently mis-expressed in cancers, potentially by genome-wide epigenetic modifications that often accompany carcinogenesis or by misexpression of meiosis-specific factors [3,4]. As the testis is immune privileged, the immune system does not recognize CTAs as self-proteins. Expression of CTAs outside of immune privileged sites can induce an immune response, thereby enabling a cancer-specific immune response to CTAs. These observations support the rationale that CTAs expressed in cancers constitute promising targets for immunotherapy. Immune targeting of CTAs could activate a long-term response against CTA-expressing cancer cells with minimal side effects on normal tissue. Since their characterization, there has been growing interest in developing immune therapeutics against CTAs [5]. Clinical trials targeting CTAs are ongoing despite some early setbacks [6–10]. Recently, it was recognized that apart from being immune targets, some CTA/CTGs can be drivers of carcinogenesis and their expression could be selected for during tumor evolution independent of the immune system [11].

As the name CTA/CTG implies, the field historically focused on the male germline. However, expression of germline-specific proteins in females is equally important, especially in the context of this review, as general homologous recombination (**HR**) factors are expected to be important for male and female meiosis alike. Thus, while we use the established nomenclature (cancer testis antigen/gene), we imply the broader sense of cancer germline antigen/genes.

In terms of their neoplastic potential, CTA/CTGs may engage in diverse mechanisms in somatic cells [11]. One prominent class of CTA/CTGs appears to lead to genome instability by impeding DNA repair, especially proteins involved in HR. Defects in HR, as exemplified by loss of BRCA1 or BRCA2 function, are associated with increased risk of breast, ovarian, prostate, pancreatic, and other cancers [12–15]. Only a small fraction (~5%) of cancers have hereditary defects in *BRCA1* or *BRCA2* [16]. A much larger number of tumors appears to exhibit a **BRCAness** phenotype, mimicking BRCA1 or BRCA2 loss, which is characterized by defects in HR caused by additional mechanisms [17]. Emerging evidence discussed in this review suggests that one mechanism to induce BRCAness is the misexpression of CTA/CTGs, which may interfere with the normal functions of BRCA1, BRCA2 or other HR proteins.

**Fig. 1.**
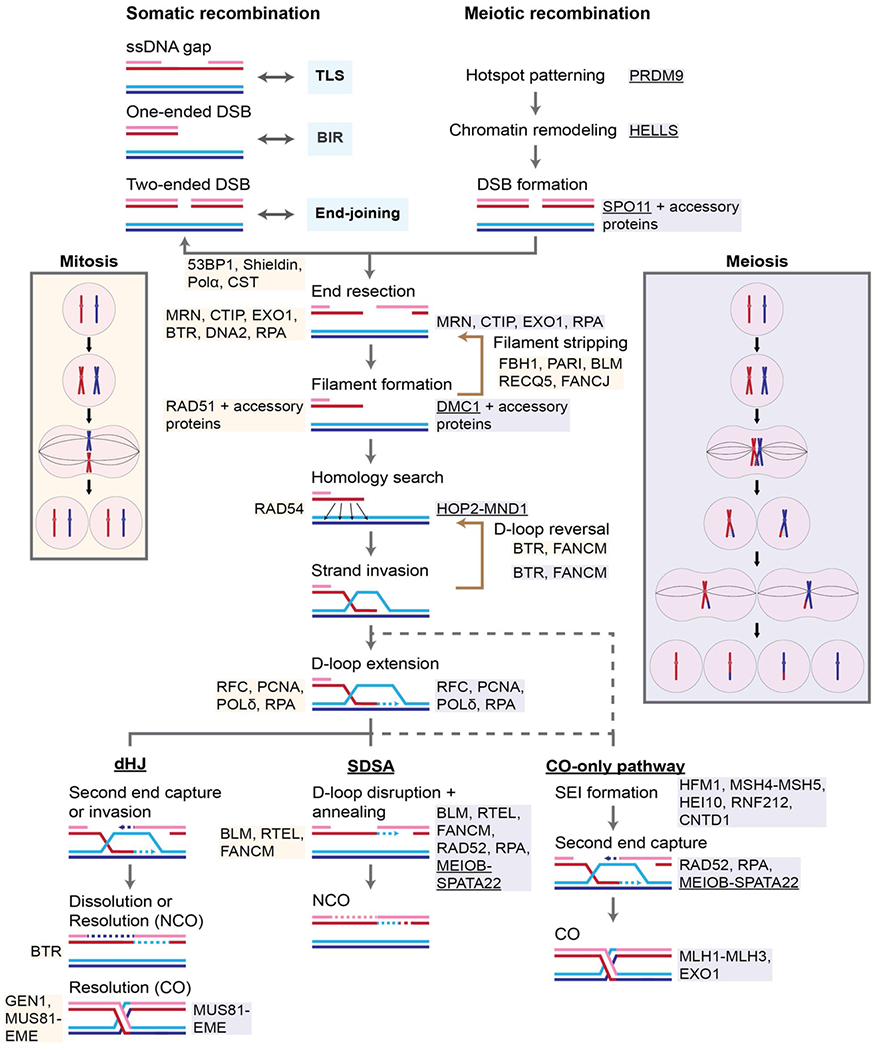
Somatic and meiotic homologous recombination pathways. Use of the somatic recombination is triggered by several types of DNA damage, including ssDNA gaps, one-ended DSBs, arising from replication fork collapse or nicks in the replication template, and two-ended DSBs. In contrast, the meiotic recombination pathway is initiated by programmed two-ended DSBs induced by SPO11 that remains covalently attached to the 5’-end of the DSB. The somatic recombination pathway competes with other DNA damage repair/tolerance pathways in the cell (*e.g.*, translesion DNA synthesis (TLS) for repair of ssDNA gaps, break-induced replication (BIR) for repair of one-ended breaks, and end-joining pathways (non-homologous end-joining, microhomology-mediated end-joining) for repair of two-ended breaks, whereas alternative repair pathways are repressed during meiosis to promote use of the meiotic recombination pathway. Once initiated, somatic and meiotic recombination transition through similar steps, beginning with end resection. Proteins involved at each step in the recombination pathways are indicated (yellow boxes, somatic recombination; blue boxes, meiotic recombination). Note that many proteins function in both pathways, including RAD51 and its accessory factors, which act in conjunction with DMC1 during meiotic recombination. In yeast, the function of Rad51 changes from being the sole DNA strand exchange protein in mitotic cells to being an accessory factor to Dmc1 during meiotic HR [51]. Resolution of the recombination intermediate can follow one of the three pathways indicated: a pathway that passes through a double Holiday junction (dHJ) intermediate to form crossovers (CO) and non-crossovers (NCO); a synthesis-dependent strand annealing (SDSA) pathway that gives rise exclusively to NCOs; and a meiosis-specific pathway that produces only COs. The SDSA pathway predominates during somatic recombination, whereas both the CO-only and SDSA pathways are common outcomes during meiosis. In both somatic and meiotic recombination, use of the dHJ pathway that results in both COs and NCOs is a minor pathway. The meiosis-specific CO-only pathway first transitions through a meta-stable intermediate called the single-end invasion (SEI), which is pre-destined to form a CO by forming a dHJ intermediate that is formed by second-end capture. The exact structure of the SEI and whether it includes newly synthesized DNA is unknown. Boxes depict chromosome segregation during the mitotic and meiotic cell cycles, respectively. Underlined protein names indicate that the factor has been identified as a CTG (see Table 1). Additional Abbreviations: CST, CTC1-STN1-TEN1; BTR, BLM-TOPOIIIα-RMI1/2.

The BRCAness phenotype in tumors is commonly defined by genomic scars, classified as mutational signatures, of which at least five are caused by defects in *BRCA1* or *BRCA2* [16–19]. While exceedingly useful as a molecular tumor classification tool, genome sequence-based approaches may not report fully accurately on the actual HR-status of tumor cells, as HR-deficiencies are known to revert but retain their genomic scars [20–22]. Alternatively, a cytological assay to assess HR-status measures DNA damage-induced focus formation and focus turnover of RAD51, a central HR protein [23–25]. RAD51 foci may represent RAD51 filaments on ssDNA, joint molecule intermediates, or duplex DNA. This approach can define functional HR status but will likely underestimate the extent of HR-deficiency, as RAD51 focus formation and turnover does not report on the entire HR process, especially not on the later steps of HR-associated DNA synthesis and joint molecule resolution/dissolution [26] (Fig. 1). Hence, additional biomarkers that report on HR status are needed, and protein level expression of HR inhibitors, such as certain CTA/CTGs that cause HR defects, could be an independent and complementary approach to detect HR deficiency in tumors.

**Fig. 2.**
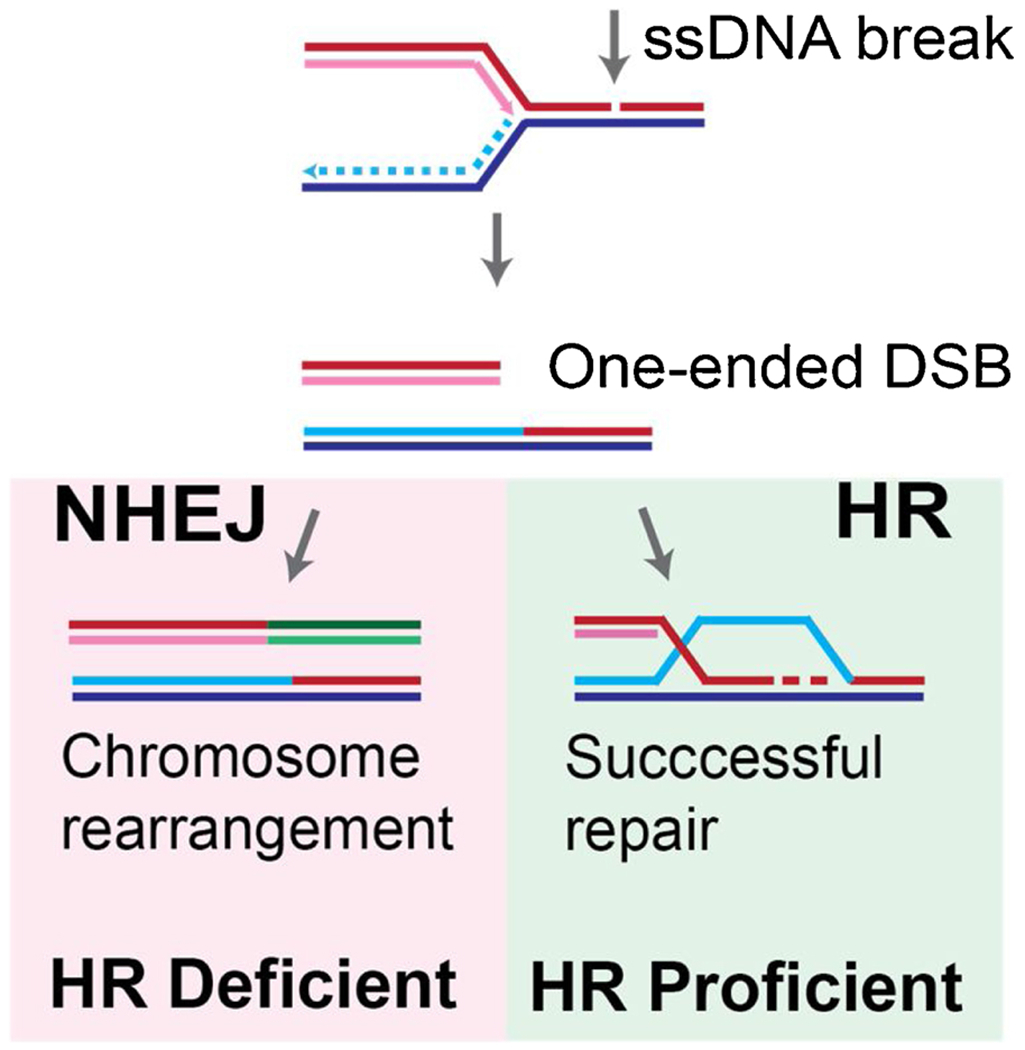
Replication-dependent formation and recovery of one-sided DNA double-stranded breaks during S-phase. Recovery of one-sided DSBs by homologous recombination (**HR**) in HR-proficient cells. In HR-deficient cells, the recovery is by non-homologous end-joining (**NHEJ**) which is joining the single end DSB to an ectopic DSB resulting in chromosome rearrangements.

This review will highlight recent research identifying CTA/CTGs that normally function in meiotic chromosome biology and DNA metabolism with special emphasis on DNA repair by HR (Table 1). Tumors deficient in HR are sensitive to targeted molecular therapies such as poly (ADP-ribose) polymerase (**PARP**) inhibitors [27–30]. PARP functions in the repair of ssDNA breaks that can be caused as byproducts of DNA damage and during DNA repair. PARP inhibitors trap PARP at the site of DNA damage and block the repair of ssDNA breaks, causing them to convert to one-ended DNA double-stranded breaks (**DSB**) during DNA replication. During S-phase, such breaks can only be productively repaired by the HR pathway, whereas immediate end-joining would result in genomic rearrangements (Fig. 2). This provides a rationale why HR-defective cells are hypersensitive to PARP inhibitors. Thus, CTA/CTGs that lead to HR defects could serve as biomarkers to make patients eligible for treatment with PARP inhibitor therapy. We evaluate this rationale by discussing published studies and results from relevant public databases to analyze expression and co-expression patterns of CTA/CTGs, their genetic and epigenetic regulation, the dependency of cancer cells on CTA/CTG expression, as well as their relationship to known driver mutations. Lastly, we discuss how future studies could maximize our knowledge of CTA/CTGs and their roles in maintaining genomic instability.

## 2. A repertoire of cancer testis (cancer germline) genes and antigens

Identification of CTAs began with the discovery of an X-linked protein, Melanoma Antigen Gene Family-A1 (MAGE-A1), as a tumor-specific antigen recognized by T cells in human melanomas [31]. Following this breakthrough, numerous tumor antigens, including another X-linked protein, NY-ESO-1 (also known as CTAG1B) [32], were found to be expressed in testes and termed cancer testis antigens (CTA) [5]. Many of these early CTAs are X-linked multi-copy genes that are located in rapidly evolving palindromic sequences of the X chromosomes, and underwent distinct evolution between rodent and primate lineages, including humans [33,34]. Expression of these CTAs is predominantly in the testis, but some are also expressed in the placenta, brain, and embryonic ovaries [35,36]. Among these X-linked CTAs, the MAGE family proteins and NY-ESO-1 attracted particular attention as potential targets of immunotherapy.

Although initially CTAs were defined as tumor-specific antigens, later studies using genomic approaches identified an increasing number of testis-specific genes that are ectopically expressed in cancers as cancer testis genes (**CTGs** or cancer germline genes) without direct validation as tumor-specific antigens [36–38]. Based on these findings, a manually curated public database recorded 204 genes as CTGs as of 2009 (http://www.cta.lncc.br) [2]. Notably, half of these CTGs are X-linked genes (termed CT-X genes), and the other half are autosomal-linked single-copy genes (non-CT-X genes). The advent of next-generation sequencing facilitated identification of additional CTGs: one study identified 1,019 CTGs [39] and another studies identified 1,103 CTGs [40]. These genome-wide analyses increased the number of single-copy non-CT-X genes, of which many are evolutionarily conserved germline genes, but many await validation as tumor-specific antigens.

The systematic analysis of various cancer types provided a clue about the regulation of CTGs in various cancers. Only a few CTGs (typically between 0 and 2) were highly expressed in a given tumor, indicating that there is generally no wide-scale misexpression of germline proteins or implementation of a germline transcriptional program in cancers [39]. Genome-wide analyses found that demethylated promoters were often located proximally upstream of testis-specific genes [39] and that CTA activation correlated with global hypo-methylation [41], supporting the notion that DNA demethylation maybe a key process in the activation of CTA/CTGs [3]. Indeed, DNA methylation is important for the regulation of germline genes (summarized in [42]). Promoters of germline genes undergo demethylation in primordial germ cells [43,44], and promoter methylation levels of most of the annotated genes are largely unchanged (mostly hypomethylated) during postnatal spermatogenesis [45]. However, the expression of CTGs is subject to stage-specific changes in spermatogenesis; in particular, CT-Xs are subject to meiotic sex chromosome inactivation and post-meiotic activation [33,34]. Thus, promoter hypomethylation maybe a prerequisite for the activation of CTA/CTGs, but it is not the only mechanism for gene activation in spermatogenesis. Of note, many testis-specific non-coding RNAs were found to be associated with CTG expression in various cancers, raising the possibility that non-coding RNAs regulate CTG expression [39,46]. CTGs could be either activated (*e.g.* ncRNA LINC00577 activates CTG *LIN28B* expression) or inhibited (*e.g.* ncRNA LINC00254 inhibits CTG *MEIOB* expression) by the expression of nearby testis specific ncRNAs [39].

In conclusion, genome-wide analyses significantly expanded the universe of CTA/CTG candidates providing a rich source for studies of their role in carcinogenesis and as potential therapeutic targets and biomarkers.

## 3. Homologous recombination and its functions in somatic and meiotic cells

The mitotic and meiotic divisions both give rise to daughter cells following chromosome replication, but the two pathways are mechanistically distinct from one another (Fig. 1). The mitotic program consists of a single, equational division, giving rise to two daughter cells that are genetically identical to the parent cell. This mode of cell division is typically utilized as a mechanism of proliferation. In contrast, the meiotic program is made up of two successive divisions: a reductional division (meiosis I) that segregates the maternal and paternal homologs, followed by an equational division (meiosis II) that segregates sister chromatids, similar to the mitotic program, resulting in an overall halving of ploidy. Meiosis generates the haploid gametes that are required for biparental reproduction. In addition, meiosis reassorts genetic information from the two parents, thereby producing daughter cells that are genetically distinguishable from the parental cells [47].

A critical feature of the meiotic program is the first meiotic division. This reductional division is achieved through the pairing and disjunction of homologous chromosomes from the mother and the father toward opposite poles. Two important modifications of the mitotic chromosome segregation program are required for the first meiotic division. First, the kinetochores of each pair of sister chromatids must attach to spindle microtubules emanating from the same pole. This is achieved through a structural modification of the sister kinetochores by the monopolin protein. Additionally, the homologous chromosomes must become physically linked to one another to enable their stable biorientation on the meiosis I spindle. These links are formed *via* crossover (**CO**) formation between homologous chromosomes [47] (Fig. 1). The process depends on meiosis-specific cohesins, including the CTGs *REC8, STAG3*, and *SMC1β* (Table 1). For a more comprehensive discussion of this process, readers are referred to review articles focused on the topic of meiotic recombination [47,48].

Meiotic recombination is a unique and highly specialized form of HR, and we emphasize here the differences between somatic and meiotic recombination [47–49] (See Fig. 1 and its legend for the mechanistic steps in the HR pathway.) Meiotic recombination utilizes many of the factors involved in the somatic recombination pathway as well as meiosis-specific components, but does not constitute an independent HR pathway. Instead, meiotic HR represents a cooption of somatic recombination as a means to physically link the homologous chromosomes (Fig. 1). Hence, meiosis-specific HR proteins interact with somatic HR proteins and function to bias HR product formation away from inter-sister non-crossover (NCO) events toward interhomolog COs.

Meiotic recombination differs from somatic recombination in that it is initiated by programmed DSBs at predetermined sites. By comparison, somatic recombination is triggered by unscheduled DNA lesions including DSBs, ssDNA gaps, and stalled/collapsed replication forks. In addition, somatic HR competes with other somatic DNA repair pathways such as non-homologous end-joining (NHEJ), microhomology-mediated end-joining (MMEJ), and break-induced replication (BIR). Competition between the meiotic HR pathway and alternative DNA repair pathways is limited through several mechanisms, including the covalent linkage of SPO11 to the DNA following DSB formation [50]. Modification of the DNA ends by SPO11 prevents non-homologous end-joining, a major competitor with the HR pathway for repair of two-ended DSBs in somatic cells.

Another important distinction between the somatic and meiotic recombination pathways is that the somatic pathway relies on the RecA homolog RAD51 for DNA strand invasion, while the meiotic pathway requires an additional RecA homolog, DMC1, in many organisms including mammals [47–49]. In spite of their high similarity and shared DNA strand exchange activity, RAD51 and DMC1 affect distinct recombination outcomes. RAD51-dependent somatic recombination preferentially engages the sister chromatid as the repair template, thereby limiting the risk of associated homozygosis or chromosome rearrangement. In contrast, DMC1-mediated meiotic recombination is strongly biased toward interhomolog recombination (so-called “interhomolog bias”). These differences in partner choice reflect the distinct goals of the somatic and meiotic HR programs: whereas somatic recombination is a mechanism of DNA repair/DNA damage tolerance, meiotic recombination must physically link the homologous chromosomes to one another. Interactions between RAD51, DMC1, and numerous protein-specific accessory factors, including the RAD51 paralogs for RAD51 and HOP2-MND1 for DMC1, may also act to enforce these partner choice preferences. Moreover, in yeast, Rad51 acts as a Dmc1 accessory factor during meiotic HR, and Rad51 filament formation is required for wild-type Dmc1 filament formation and recombination [51]. Genome-wide chromatin-immunoprecipitation data from mouse meiosis are consistent with this interpretation [52].

Lastly, there are differences in the mechanisms through which the DNA strand exchange intermediates are matured and resolved into products. During meiosis I, at least one CO forms between each pair of homologous chromosomes, even though a majority of DSBs are repaired without crossing over. This highly regulated distribution of COs is achieved using meiosis-specific factors, which include HFM1, MSH4-MSH5, TEX11-SHOC1-SPO16, HEI10, PRR19, RNF212 and CNTD1 that stabilize interhomolog recombination intermediates, and promote their maturation into COs *via* the MutLγ endonuclease (MLH1-MLH3) (Fig. 1). While CO formation in somatic HR is generally considered to be a minority outcome [26], recent genetic analysis of the U2OS and Saos-2 human cell lines unexpectedly revealed that CO formation can also be a relatively common outcome of repair of two-ended DSBs by somatic HR [53].

Our understanding of meiotic recombination is continuously evolving. Many highly conserved meiosis-specific proteins, such as SPO11, DMC1, and REC8, have clearly established and conserved activities across budding yeast, plants, mice, and humans (Fig. 1). Yet the functions of other factors that are essential to meiotic recombination, like HOP2-MND1, have not been fully elucidated. In higher eukaryotes, meiotic HR is further complicated by the ongoing discovery of novel recombination factors that have no homologs in budding yeast. Recent examples include MEIOB, SPATA22, HSF2BP/MEILB2, and BRME1/C19orf57 [54–59], which were also identified as CTGs (Table 1). The lack of mechanistic information as to the roles of many meiotic recombination proteins limits our ability to understand how they could interfere with somatic HR when mis-expressed in somatic cells.

In summary, meiotic recombination is a specialized type of HR that functions to physically link the homologous chromosomes to one another to promote their reductional segregation at meiosis I. Importantly, meiosis-specific HR proteins cooperate with general HR factors that also function during DNA damage repair/tolerance in somatic cells.

## 4. Mis-expressed proteins involved in meiotic chromosome metabolism and their role in carcinogenesis

The genome-wide analyses significantly expanded the numbers of CTA/CTG candidates, but their relationships to cancer largely remains to be determined. To start closing this knowledge gap, we conducted highly focused database analyses using the proteins listed in Table 1, which have known roles in meiotic chromosome biology and HR.

There are many similarities between testicular germ cells and cancers such as CTA/CTG expression, hypoxic environments, metabolic states, and reductional division that can cause homozygosis and chromosome rearrangement [60–62]. It has been proposed that CTA expression in somatic cells could promote tumor development by leading to a germ-cell state transition that is beneficial to tumor development and growth [4,5,63]. The premise is that multiple germ cell antigens, especially those that function together in meiosis, are mis-expressed simultaneously in cancer cells. While genome-wide analysis has revealed that generally only a few CTA/CTGs are highly expressed in a given tumor [39], we specifically analyzed the co-expression patterns of the CTA/CTGs in Table 1 in tumors and cancer cell lines using the Metabolic gEne Rapid Visulaizer (MERAV, http://merav.wi.mit.edu) portal [64]. This platform analyzes transcriptomic data from cancers and cell lines from a variety of cancer databases and calculates the gene expression correlation. Our findings indicate that multiple CTA/CTGs, especially those that function as a complex or in the same pathway in germ cells, are often not co-expressed in cancer cell lines and tumors (Suppl. Fig. 1). For example: in germ cells, MEIOB and SPATA22 form a complex which interacts with RPA and localizes to sites of meiotic DSBs and facilitates DNA strand exchange. However, in lung adenocarcinomas expression of *MEIOB* and *SPATA22* is mutually exclusive [39]. Hence, it appears unlikely that cancer cells expressing these germline antigens are undergoing a programmed transition to a germ cell state.

CTGs are classified as testis-selective (expression in testis and a few somatic tissues) or testis-restricted (expression only in testis) [65]. We analyzed gene expression patterns across tumors and normal tissues using the Gene Expression Profiling Interactive Analysis (GEPIA; http://gepia.cancerpku.cn) database for the genes listed in Table 1 along with a small cohort of somatic HR proteins for comparison [66] (Suppl. Fig. 2). Our analysis reveals that most CTGs are expressed in a variety of normal somatic tissues in addition to being highly expressed in testis (Suppl. Fig. 2). When comparing the expression levels of the CTGs from Table 1 with the expression of somatic HR genes including *RAD51, RAD54, RAD51AP1, RAD21* and *SMC1α* across various normal tissues and cancers (Suppl. Fig. 2), we identified highly variable expression with few recognizable patterns. 1) Testicular germ cell tumors (**TGCTs**) have reduced expression of CTGs and meiotic entry regulator genes, whereas RNA levels of the selected somatic HR proteins appear to be higher in TGCT than in normal testis (Suppl. Fig. 2 and [4]). 2) Many CTGs listed in Table 1, such as *DMC1, HELLS, HOP2, MND1, HFS2BP, REC8, STAG3, SYCP2*, and *SYCE1* show appreciable RNA-level expression in normal tissues outside testis, some in the range of somatic HR factors. 3) Other CTGs, such as *PRDM9, RAD21L, SPO11, SSX1, SYCP1, SYCP3, TEX12*, and *TEX19* show little if any RNA-level expression in normal tissues outside testis. It is unclear how well the RNA levels correlate with protein levels. There are limited CTG protein level expression data available, and they are also subject to variations in detection sensitivity and expression cutoff [39]. Protein expression data in somatic tissues and cell lines are available for some CTGs listed in Table 1 including REC8, DMC1, SPO11, HORMAD1 and HOP2, and show that their protein expression is not strictly specific to the germline [67–71]. The functional significance of the expression of these germline proteins in normal somatic tissues is unknown.

Gene amplification is a common mechanism for mis-expression of genes, and we explored if CTG expression is correlated with gene copy number amplification. For the selected CTGs of Table 1, there is weak to no apparent correlation between CTG expression and gene copy number amplification (Suppl. Fig. 3). The gene encoding epidermal growth factor receptor (EGFR) was utilized as a positive control, and none of the CTGs reached that level of correlation. This analysis was not resolved for tumor type, explaining the relatively low Pearson’s coefficient for *EGFR*. Among CTGs, *SYCP2* and *TEX12* showed the highest Pearson’s coefficients, and tumor type-resolved analysis did reveal significant correlation between RNA levels and gene copy number for these and additional CTGs in certain tumor types (Suppl. Table 1). We conclude that gene amplification is one but not the only mechanism underlying mis-expression of certain CTGs of Table 1 in specific tumor types.

Cancer cells can become dependent (‘addicted’) on the expression of normally non-essential genes, which may identify cancer-specific vulnerabilities. Some genes involved in maintaining genome stability are essential, for example *BRCA1* and *BRCA2*, while other HR genes such as *RAD54* or *BLM* are not (for detailed information see [26]). Cancer gene dependency is defined as a gene whose expression is required for the proliferation or survival of cancer cells [72,73]. We used the Dependency Map (DepMap; https://depmap.org/portal) database to analyze the dependency of cancer cell lines on the CTGs listed in Table 1 focusing on the CRISPR knockout data as they tend to be more robust than the siRNA datasets [74]. Cancer cell lines generally showed low to no dependency on the CTGs (Suppl. Table 2). We have included dependency data for the prototypical oncogene *MYC* as a positive control, and 99% of the cell lines are dependent on *MYC* (Suppl. Table 2). There were some cell lines that had moderate dependency on a CTA/CTG (see Suppl. Table 2 and other reported dependencies [68,75–77]). Such dependencies appeared to be cell line-specific and did not apply to a specific cancer type. We infer that, when expressed, CTA/CTGs do not generally assume an essential function in cancer cells. This is consistent with the overall conclusion that CTGs listed in Table 1 do not exert their regular function when misexpressed in cancer cells but rather interfere with normal HR-mediated repair or otherwise cause genomic instability (see 6. Concluding remarks). The discrepancies between the database analyses and published studies may be technical (CRISPR *vs.* RNAi; differently scored endpoints). Though CRISPR-knockout is more rigorous than siRNA knockdown, tumors are extremely adaptable, so the DepMap analysis may miss important changes occurring during tumor proliferation. There is a need for further studies of CTGs and their expression in specific tumor types to fully understand the effects of CTGs on tumor growth and proliferation.

Mutually exclusive CTG expression with inactivating mutations in the major HR genes, *BRCA1* and *BRCA2*, that drive tumor formation may indicate functional significance. Using genomic and transcriptomic breast cancer data [78,79], we found that tumor cell expression of the CTGs listed in Table 1 to be seemingly mutually exclusive of inactivating mutations in *BRCA1* and *BRCA2*, although the results were not statistically significant due to low sample size of tumors containing *BRCA1/BRCA2* mutations and CTG overexpression. A published study revealed mutually exclusive expression of CTGs including *MEIOB* (Table 1) with mutations in *PIK3CA*, one of the most frequently mutated oncogenes in human cancers, in a statistically significant manner [39]. Our analysis of the Table 1 CTGs identified statistical significance for *DMC1* and *HSF2BP* expression being mutually exclusive of *PIK3CA* mutations (not shown). These analyses are currently limited by low sample sizes, and there is a clear need for more cancer samples with HR gene mutations and CTA overexpression to achieve statistical significance.

Apart from DNA repair by HR, CTA/CTGs can also promote oncogenesis by affecting other pathways such as transcription (*TEX19* [80]), mutagenic DNA damage tolerance by translesion DNA synthesis (*MAGE-A4* [81]), or as oncogenes (*SYCP3* [76,82], *HELLS* [83]) and tumor suppressors (*REC8* [84,85]) (Table 1). While non-CT-Xs have specific functions in DNA metabolism (Table 1), CT-Xs tend to be intrinsically disordered proteins, lacking rigid 3D structure or enzymatic functions, and their functions in germ cells remain largely unknown [86]. CT-Xs like *MAGE-A4* and the *SSX* family of proteins are not seemingly involved in DNA metabolism in germ cells but have been shown to affect DNA repair and genome stability when expressed in somatic cells [81,87] (Table 1). A recent study demonstrated that *MAGE* genes evolved to protect the mammalian male germline against environmental stress, and proposed that cancer cells exploit *MAGE* genes to facilitate their cell growth [88]. Thus, certain CTAs may increase the fitness in reproduction and cancer survival.

In conclusion, the CTA/CTGs listed in Table 1 are rarely co-expressed with their meiotic partner proteins in cancer cells. Hence these proteins are unlikely to be performing their normal meiotic function in somatic cells. CTA/CTGs that are mis-expressed in cancers have varying functions in meiosis. They include structural components of the synaptonemal complex, recombinases, HR accessory proteins and cohesins. In germ cells, meiotic proteins interact with somatic HR proteins to achieve specialized functions, including interhomolog recombination and crossing over. So, it is likely that when a given CTA/CTG is expressed in somatic cells, it will similarly interact with its somatic recombination partners. However, since other meiotic interaction partners are unavailable, such interactions lack their regular environment and could lead to dysfunction and genome instability. For example, immune histochemical analysis of 52 triple-negative breast cancers and 32 adjacent tissues found that *MEIOB* was significantly upregulated in the tumors [89]. Overexpression of *MEIOB* in the SUM1315MO2 breast cancer cell line led to significantly decreased γH2AX foci in response to cisplatin treatment; however, HR measured in a GFP-reporter assay was also decreased. These findings suggest that there may be an early defect in γH2AX focus formation in response to *MEIOB* expression in the SUM1315MO2 cell line, possibly as a result of aberrant association between MEIOB and its paralogue, RPA.

## 5. Potential mechanisms how CTA/CTGs may affect may affect genome stability

**Fig. 3.**
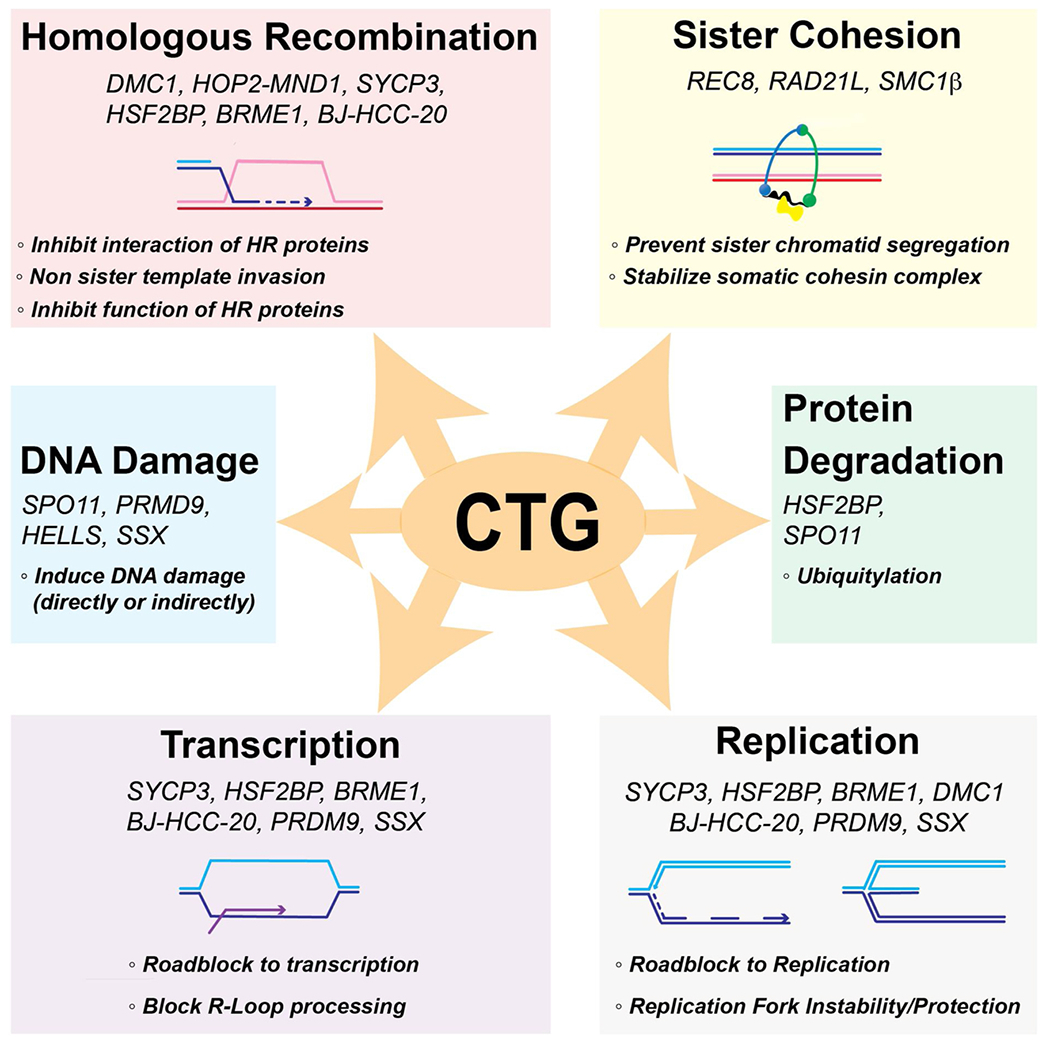
Possible mechanisms by which cancer testis genes with a function in meiotic chromosome metabolism may affect genome stability. See Table 1 for the list of proteins, their normal meiotic functions, and their proposed effects during in somatic cells.

Fig. 3 illustrates a number of postulated and potential mechanisms for how the CTA/CTGs listed in Table 1 could interfere with genomic stability. Table 1 summarizes the key observations along with the relevant primary literature. While no particular mechanism has been firmly established, a few themes appear to be crystalizing from these initial analyses.

### 5.1. Homologous recombination

Defects in HR are associated with and causative of strong meiotic phenotypes. However, not all CTA/CTGs that are required for meiosis affect HR directly. Thus, whether a protein, whose loss causes male/female infertility due to a meiotic HR defect, is directly involved in the HR process and possibly disruptive of HR in somatic cells has to be established through careful mechanistic work. The HR process is a highly complex pathway involving dozens of proteins that are required in specific stoichiometries. Misexpression of a single component that is capable of physically interacting with somatic HR proteins, such as DMC1, HOP2-MND1, SYCP3, REC8, MEIOB, SPATA22, and HSF2BP (see Table 1, Fig. 3), has the potential to interfere with normal functioning of the process, as discussed for the example of MEIOB above [89]. Moreover, meiotic components may affect template choice (sister chromatid vs. homolog) during homology search and DNA strand invasion leading to pathological interhomolog events in somatic cells.

### 5.2. Sister cohesion/centromere dysfunction

Misexpression of a single cohesin component, such as the meiosis-specific kleisin *REC8* or other cohesion components such as *STAG3*, or *SMC1β* (see Table 1, Fig. 3), may interfere with timely cohesin cleavage and sister chromatid segregation. Somatic expression of *REC8* (and *SPO11*) in fission yeast and human cells unexpectedly led to dysfunctional kinetochores through eviction of CenpA containing nucleosomes involving specific ATP-dependent chromatin remodelers [90].

### 5.3. DNA damage

Several CTGs have the potential to directly or indirectly induce DNA damage, such as SPO11, or possibly by their activity on chromatin, such as PRDM9, HELLS and SSX (see Table 1, Fig. 3). For example, in a comprehensive analysis of over 1,500 cancer samples across 39 cancer types, PRDM9 was found to be misexpressed in ~20% of tumors [91]. Interestingly, structural variant breakpoints in these tumors were significantly enriched at PRDM9 recognition sites. Similar to the results of our database analysis, the authors found no evidence that SPO11 was co-expressed with PRDM9, arguing against a model wherein PRDM9 is directly responsible for DSB formation in these tumors. It is possible that DNA binding by PRDM9 is interfering with transcription and replication to induce DNA damage indirectly (see next paragraph).

### 5.4. Transcription and replication

It is well established that transcription and replication cause significant DNA damage that leads to genomic instability. A number of CTGs, such as *BJ-HCC-20, BRME1, DMC1, HFS2BP, PRDM9*, and *SSX* (see Table 1, Fig. 3), that encode DNA binding proteins and interact with components of the transcriptional/replication machinery including proteins in the pathways for processing of R-loops and stalled or broken replication forks have the potential to increase genomic instability [56, 59,68,77,87,91,92]. However, specific mechanisms still need to be elaborated.

### 5.5. Protein degradation

Expression of CTGs may cause degradation of central HR proteins. One such example is *HSF2BP*, which triggers the degradation of BRCA2 when expressed in somatic cells [77] (see Table 1, Fig. 3).

In sum, there is a growing body of data implying that the expression of a single CTA/CTG in somatic cells can interfere with somatic processes. Numerous initial studies have documented hallmarks of genomic instability, including hyper-sensitivity to DNA damaging compounds, in cells misexpressing the proteins listed in Table 1.

## 6. Concluding remarks

On the basis of our literature review and the database analyses presented, we reach the following important conclusions:

1. There are possibly significantly more CTA/CTGs than reported previously, especially when considering that more research is needed on the female germline. The status as cancer antigens has often not been validated. CTGs encode a diverse group of proteins, only some of which are exclusively expressed in germ cells. CTGs with expression outside the testis might not be expected to be antigenic. CT-Xs, including many traditional CTAs, and non-CT-Xs, including many newly-discovered CTGs, encode distinct groups of proteins subject to different regulation based on the chromosomal locations of their genes. Moreover, CTA/CTGs have diverse meiotic functions and thus evoke differential impacts on somatic cells when misexpressed. Further analysis of female meiosis is warranted to establish the complete repertoire of cancer germline antigens/genes that are directly involved in meiotic recombination.
2. Concerted misexpression of numerous CTA/CTGs is rarely observed. This finding argues against the germ cell theory of cancer. Furthermore, though many meiotic proteins function within a heterodimer or protein complex (*e.g.* HOP2-MND1) that can also include constitutive proteins (*e.g.* MEIOB and SPATA22 form a complex with RPA; BRME1 and MEILB2 form a complex with BRCA2), CTGs are seldom co-expressed with their interaction partners. Thus it is unlikely that these CTGs are proficient to carry out their meiotic function in somatic cells. Instead, we propose that the abilities of these CTG encoded proteins to bind DNA and interact with somatic HR proteins interfere with DNA replication, mitotic sister chromatid segregation, and somatic DNA repair (Fig. 3).
3. Misexpression of a single or several CTA/CTGs has the potential to lead to genome instability (Fig. 3). Consistent with the model that the DNA binding activities of CTA/CTGs or their interaction with somatic HR proteins can interfere with processes critical to genome stability in somatic cells, emerging evidence for several CTGs suggests that misexpression of a single CTG can lead to genomic instability (Table 1). It is not known at what expression level a particular CTG disrupts HR. More functional cell-based studies are needed to determine these levels for each CTG of interest.
4. An optimal CTA/CTG to be used as a marker for HR-deficiency in tumors or as a therapeutic target should have testis-restricted expression. However, transcriptional data indicate that many meiosis-specific genes are expressed in normal somatic tissues [65]. Yet analysis of gene expression by mRNA levels can be problematic, since there are multiple levels of post-transcriptional control in higher eukaryotes and not all mRNAs are necessarily translated into a protein product [39,93] and only limited protein expression data are available. This highlights the need for more comprehensive CTA/CTG proteomics analysis in tumors, normal tissue and cancer cell lines.
5. It may be possible to develop certain CTA/CTGs as biomarkers of cancer cell deficiencies of important cellular functions, such as HR-deficiency. This knowledge can be exploited to determine to which therapies the tumor is most likely to respond. For instance, PARP inhibitors may be especially effective in treating HR-defective tumors, as these cancers would be particularly vulnerable to additional disruptions in the recombination pathway.

## Supplementary Material

Supplementary material

## Figures and Tables

**Table 1:** Selected cancer-testis antigens/genes and their established functions in meiotic chromosome metabolism and homologous recombination with their proposed roles in carcinogenesis [39,56,59,67–70,75–77,80–85,87,89–92,94–103,105–112].

Protein	Meiotic function	Proposed role in carcinogenesis
BJ-HCC-20A	Unknown	BRCA2 interaction, promotion of cell growth and inhibition of apoptosis [92, 94]
BRME1/C19orf57	Required for DMC1 focus formation	Reduction in RAD51 focus formation [56, 59]
DMC1	Homologous recombination	Replication fork stability [68]; enable interhomolog biased recombination?
HELLS	Chromatin Remodeler	Interaction with E2F3 to promote tumor progression [83]
HOP2/PSMC3-IP-MND1	DMC1 accessory factor	Increase in chromosome mobility, telomere exchanges [70, 95]; cell cycle progression by upregulating E2F1 expression through interaction with KLF6 [75]
HORMAD1	Required for DSB formation and/or resection, SC formation	Promotes CtIP mediated resection [96] and RAD51 filament formation [97]; inhibition of HR [98]; prevents MCM8-MCM9 nuclear localization and limits MLH1 mismatch repair [99]
HSF2BP/MEILB2	Required for DMC1 focus formation	Interference with BRCA2 function and HR [77]
MAGE-A4	Transcriptional repressor	Increase in translesion DNA synthesis [81]
MEIOB	ssDNA binding protein required for meiosis I progression	Homologous recombination deficiency and genome instability [39, 89]
PRDM9	Designates locations of DSBs	Whole-genome rearrangements [91]
RAD21L	Sister chromatid cohesion	Promotes homolog alignment [100]; aberrant chromosome segregation leading to genome instability [67, 101]
REC8	Sister chromatid cohesion	Tumor suppressor [84, 85]; increased cancer cell survival by facilitating ploidy reduction in endopolyploid cells [102]; promotes loss of mitotic kinetochores [90]
SMC1β, STAG3	Sister chromatid cohesion	Increased expression in some cancers [67, 103]
SPO11	Catalytic component of complex that creates DSBs	Increased expression in certain cancers, induces DNA damage [69]; promotes loss of mitotic kinetochores [90]
SSX family	Transcriptional repressor	Genome instability [87]
SYCE1, SYCP1, SYCP2	Structural components of synaptonemal complex	Genome instability [105, 106]; expression in cancers [107, 108]
SYCP3	Lateral element of synaptonemal complex	Interferes with BRCA2, RAD51 function [109, 110]; interacts with AKT to promote tumor formation [76]; upregulates VEGF-C and VEGF-D to promote metastasis in lung cancers [82]
TEX12	Central element of synaptonemal complex	Centrosome amplification [111]

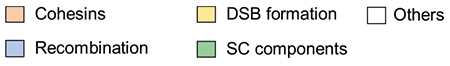

## Abbreviations:

BRCAness: cellular phenotype of HR-deficiency caused by impaired function of BRCA1, BRCA2, and other HR factors
CO: crossover
CTA: cancer testis antigen
CTG: cancer testis gene
CTncRNA: cancer testis non-coding RNA
DSB: DNA double-stranded break
HR: homologous recombination
PARP: poly (ADP-ribose) polymerase
NCO: non-crossover
ncRNA: non-coding RNA
TGCT: testicular germ cell tumor
