# Supplementary material for: Cancer testis antigens and genomic instability: More than immunology
Source: DNA Repair (Amst). Author manuscript; Available in PMC 2022 Jun 14. (PMC9196322; doi:10.1016/j.dnarep.2021.103214)
Supplement: Supplementary material [file NIHMS1811464-supplement-Supplementary_material.pdf]

**Supplement:**

# Cancer testis antigens and genomic instability: More than immunology

Ash Jay[1], Diedre Reitz[1], Satoshi H. Namekawa[1] and Wolf-Dietrich Heyer[1,2*]

[1] Department of Microbiology and Molecular Genetics,

[2] Department of Molecular and Cellular Biology,

University of California, Davis, Davis CA 95616-8665 (USA)

* Corresponding author:

Wolf-Dietrich Heyer, wdheyer@ucdavis.edu Tel. (530)752-3001 FAX (530) 752-3011

This supplement contains:

2 Supplemental Tables

3 Supplement Figures

4 Supplemental References

| Gene | Cancer subtypes where gene expression has moderate to strong positive correlation with copy number amplification |
|---|---|
| *EGFR* (positive control) | breast: 0.499; esophagus: 0.695; lung: 0.410; liver: 0.455; upper aerodigestive: 0.585; thyroid: 0.611 |
| *DMC1* | plasma cell: 0.427 |
| *HELLS* | upper-aerodigestive: 0.38; plasma cell: 0.404; skin: 0.35; pancreas: 0.332; central nervous system: 0.391 |
| *HOP2* | lymphocyte: 0.453; skin: 0.376 |
| *MND1* | urinary tract: 0.468; liver: 0.419; gastric: 0.344; breast:0.310; bile duct: 0.827 |
| *HORMAD1* | central nervous system: 0.394 |
| *HSF2BP* | kidney: 0.389; soft tissue: 0.73 |
| *MEIOB* | gastric: 0.554; pancreas: 0.380 |
| *PRDM9* | blood: 0.369; thyroid; 0.781 |
| *SMC1β* | breast: 0.401 |
| *STAG3* | central nervous system: 0.358; pancreas: 0.306; upper aerodigestive: 0.486 |
| *RAD21L* | thyroid: 0.660; soft tissue: 0.622 |
| *REC8* | central nervous system: 0.359; kidney: 0.380; pancreas: 0.425 |
| *SPO11* | Ovary: 0.442 |
| *SYCP1* | - |
| *SYCP2* | uterus: 0.577; esophagus: 0.41; skin: 0.316 upper aerodigestive: 0.399 |
| *SYCE1* | bone: 0.529 |
| *SYCP3* | - |
| *TEX12* | bile duct: 0.908;  upper aerodigestive: 0.559; lymphocyte: 0.461; breast: 0.541; skin: 0.443; plasma cell: 0.478; lung: 0.348 |
| *TEX19* | - |

**Supplemental Table 1. Tumor-type specific correlation of gene expression with copy number.** Tumor specific analysis of data in **Supplemental Figure 3** showing cancer subtypes where gene expression has moderate to high positive correlation with copy number amplification and are statistically significant (Pearson's correlation coefficient ≥0.3).

| Gene | Dependent cell lines based on CRISPR screens/Total cell lines | Example of Cell lines with dependency score ≤ -0.5 |
|---|---|---|
| *MYC* (positive control) | 966/978 | LS1034 (colorectal cancer), BT549 (breast cancer) |
| *DMC1* | 0/990 | - |
| *HELLS* | 0/978 | - |
| *HOP2* | 12/978 | HT3 (cervical cancer), NCIH1666 (lung cancer), OAW42 (ovarian cancer), TCCSUP (bladder cancer), JHU029 (head and neck cancer), WM88 (skin cancer), CHP212 (neuroblastoma) |
| *MND1* | 1/990 | JHU029 (head and neck cancer) |
| *HORMAD1* | 24/990 | CHLA57 (bone cancer), A427 (lung cancer), MC116 (lymphoma), TTC642 (rhabdoid), SCH (gastric cancer) |
| *HSF2BP* | 1/990 | GIMEN (neuroblastoma) |
| *MAGE-A4* | 1/987 | NCIH2882 (lung cancer) |
| *MEIOB* | 0/990 | - |
| *PRDM9* | 0/978 | - |
| *SMC1β* | 3/978 | SW403 (colorectal cancer), COLO800(skin cancer), YAMATO Sarcoma) |
| *STAG3* | 2/990 | SW948 (colorectal cancer), GIMEN (neuroblastoma) |
| *RAD21L* | 0/990 | AML193 (leukemia) |
| *REC8* | 36/990 | SG231 (bile duct cancer), UACC893 (breast cancer), D283MED (brain cancer), COLO230 (colorectal cancer), SCH (gastric cancer), LI7 (liver cancer), CORL105 (lung cancer), PEO4 (ovarian cancer) |
| *SPO11* | 1/990 | SNU626 (brain cancer) |
| *SSX1* | 80/975 | DU4475 (breast cancer), LS123 (colorectal cancer), JHH2 (liver cancer), EJM (myeloma), HEC1B(uterine cancer), JR (sarcoma), HCC2935 (lung cancer) |
| *SYCP1* | 2/990 | OMM1 (eye cancer), CH157MN(brain cancer) |
| *SYCP2* | 0/990 | - |
| *SYCE1* | 0/990 | - |
| *SYCP3* | 0/978 | - |
| *TEX12* | 0/990 | - |
| *TEX19* | 9/990 | SIHA (cervical cancer), GSS (gastric cancer), WM115 (skin cancer), NS (neuroblastoma), NB5 (neuroblastoma), MC116 (lymphoma) |

**Supplemental Table 2. Cell line dependency on cancer-testis antigens/genes from Table 1.** The analysis of cancer cell line dependency used the Dependency Map (DepMap; https://depmap.org/portal) database [1]. Dependent cell lines have probability of dependency (probability that the dependency score is from the distribution of essential gene scores rather than non-essential gene scores) greater than 0.5 [2], and column 2 lists the number of cell lines that meet this criterion. The gene dependency scores reflects the dependency of a given cell line on a particular gene. A dependency score of 0 means the gene is not dependent, while a score of -1 indicates strong inhibition of cell survival and proliferation in the corresponding cell line. The table uses the common cutoff of ≤-0.5 in column 3.

Cohesins DSB formation Recombination SC components Others

| | DMC1 | HELLS | HOP2 | HORMAD1 | HSF2BP | MEIOB | MND1 | PRDM9 | REC8 | SMC1b | SPO11 | SSX1 | STAG3 | SYCE1 | SYCP1 | SYCP2 | SYCP3 | TEX12 | TEX19 |
|---|---|---|---|---|---|---|---|---|---|---|---|---|---|---|---|---|---|---|---|
| DMC1 | 1 | 0.44 | 0.32 | 0.01 | 0.11 | 0.07 | 0.52 | -0.07 | 0.05 | 0.08 | -0.1 | 0.08 | 0.09 | -0.01 | -0.02 | 0.02 | 0.09 | 0.05 | 0.11 |
| HELLS | 0.44 | 1 | 0.58 | 0 | 0.26 | 0.06 | -0.03 | -0.09 | 0.07 | 0.07 | -0.18 | 0.03 | 0.26 | -0.01 | 0 | -0.02 | 0.14 | 0.03 | 0.05 |
| HOP2 | 0.32 | 0.58 | 1 | -0.1 | 0.3 | 0.1 | 0.44 | -0.1 | 0.06 | 0.04 | -0.26 | 0.08 | 0.06 | -0.07 | 0.03 | -0.05 | 0.14 | -0.05 | 0.05 |
| HORMAD1 | 0.01 | 0 | -0.01 | 1 | 0.08 | 0.02 | -0.04 | -0.05 | -0.07 | 0.03 | -0.06 | 0 | -0.03 | 0.05 | -0.01 | 0.05 | 0.01 | -0.05 | 0.04 |
| HSF2BP | 0.11 | 0.26 | 0.3 | 0.08 | 1 | 0.07 | 0.14 | -0.06 | -0.11 | 0.01 | -0.24 | 0.02 | -0.04 | 0.04 | 0 | 0.05 | 0.05 | -0.13 | 0.01 |
| MEIOB | 0.07 | 0.06 | 0.1 | 0.02 | 0.07 | 1 | 0.05 | -0.03 | 0 | 0 | -0.07 | 0 | 0 | 0.09 | 0.1 | 0.11 | 0.01 | -0.02 | -0.01 |
| MND1 | 0.52 | 0.57 | 0.44 | -0.04 | 0.14 | 0.05 | 1 | -0.01 | 0.03 | 0.12 | -0.14 | 0.05 | 0.16 | -0.03 | 0.02 | -0.05 | 0.07 | 0.16 | 0.14 |
| PRDM9 | -0.07 | -0.09 | -0.1 | -0.05 | -0.06 | -0.03 | -0.01 | 1 | 0.05 | 0.1 | 0.1 | -0.03 | 0.13 | 0.01 | -0.03 | -0.02 | -0.03 | 0.2 | 0.01 |
| REC8 | 0.05 | 0.07 | 0.06 | -0.07 | -0.11 | 0 | 0.03 | 0.05 | 1 | -0.02 | 0.13 | 0.11 | 0.15 | -0.01 | 0.02 | -0.05 | -0.04 | 0.07 | -0.02 |
| SMC1b | 0.08 | 0.07 | 0.04 | 0.03 | 0.01 | 0 | 0.12 | 0.1 | -0.02 | 1 | -0.04 | 0.02 | 0.14 | 0.01 | -0.01 | 0.09 | 0.03 | 0.09 | 0.01 |
| SPO11 | -0.1 | -0.18 | -0.26 | -0.06 | -0.24 | -0.07 | -0.14 | 0.1 | 0.13 | -0.04 | 1 | -0.04 | 0.17 | -0.02 | 0 | 0 | -0.04 | 0.12 | -0.02 |
| SSX1 | 0.08 | 0.03 | 0.08 | 0 | 0.02 | 0 | 0.05 | -0.03 | 0.11 | 0.02 | -0.04 | 1 | 0.03 | 0.01 | -0.01 | -0.03 | 0.04 | -0.01 | 0.23 |
| STAG3 | 0.09 | 0.26 | 0.06 | -0.03 | -0.04 | 0 | 0.16 | 0.13 | 0.15 | 0.14 | 0.17 | 0.03 | 1 | -0.01 | 0 | 0.02 | 0.03 | 0.15 | 0.02 |
| SYCE1 | -0.01 | -0.01 | -0.07 | 0.05 | 0.04 | 0.09 | -0.03 | 0.0` | -0.01 | 0.01 | -0.02 | 0.01 | -0.01 | 1 | -0.01 | 0.05 | -0.02 | 0 | 0.03 |
| SYCP1 | -0.02 | 0 | 0.03 | -0.01 | 0 | 0.1 | 0.02 | -0.03 | 0.02 | -0.01 | 0 | -0.01 | 0 | -0.01 | 1 | -0.01 | 0.01 | 0.05 | 0 |
| SYCP2 | 0.02 | -0.02 | -0.05 | 0.05 | 0.05 | 0.11 | -0.05 | -0.02 | -0.05 | 0.09 | 0 | -0.03 | 0.02 | 0.05 | -0.01 | 1 | -0.02 | -0.02 | -0.02 |
| SYCP3 | 0.09 | 0.14 | 0.14 | 0.01 | 0.05 | 0.01 | 0.07 | -0.03 | -0.04 | 0.03 | -0.04 | 0l04 | 0.03 | -0.02 | 0.01 | -0.02 | 1 | 0.01 | 0.02 |
| TEX12 | 0.05 | 0.03 | -0.05 | -0.05 | -0.13 | -0.02 | 0.16 | 0.2 | 0.07 | 0.09 | 0.12 | -0.01 | 0.15 | 0 | 0.05 | -0.02 | 0.01 | 1 | 0 |
| TEX19 | 0.11 | 0.05 | 0.05 | 0.04 | 0.01 | -0.01 | 0.14 | 0.01 | -0.02 | 0.01 | -0.02 | 0.23 | 0.02 | 0.03 | 0 | -0.02 | 0.02 | 0 | 1 |

**Supplemental Figure 1: Correlation of cancer-testis gene (CTG) expression across cancer cell lines and tumors.** Pearson correlation using the Metabolic gEne RApid Visualizer (MERAV, http://merav.wi.mit.edu) portal expresses the association between expression of the CTGs from **Table 1** [3].

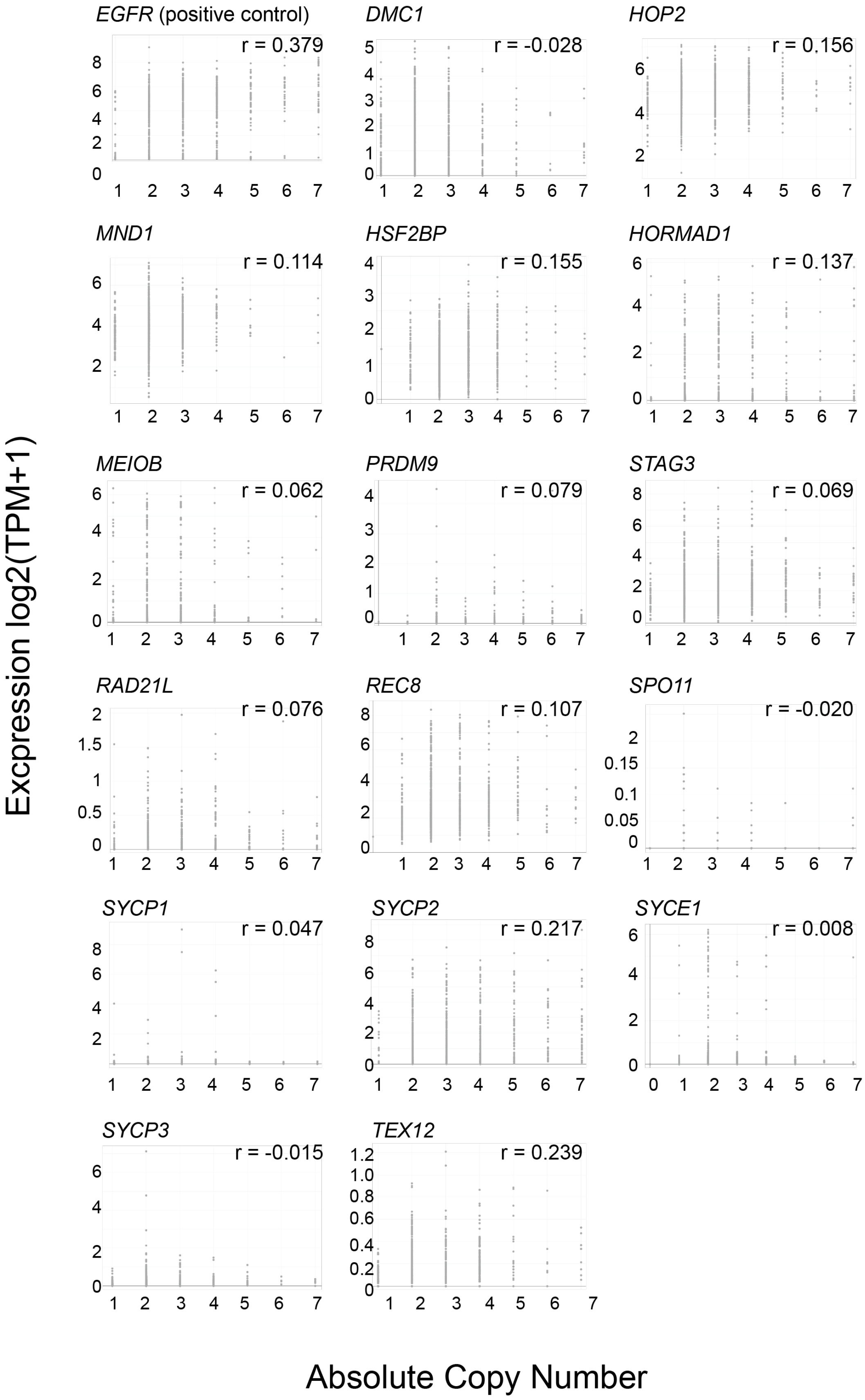
EGFR (positive control)
r = 0.379
DMC1
r = -0.028
HOP2
r = 0.156
MND1
r = 0.114
HSF2BP
r = 0.155
HORMAD1
r = 0.137
MEIOB
r = 0.062
PRDM9
r = 0.079
STAG3
r = 0.069
RAD21L
r = 0.076
REC8
r = 0.107
SPO11
r = -0.020
SYCP1
r = 0.047
SYCP2
r = 0.217
SYCE1
r = 0.008
SYCP3
r = -0.015
TEX12
r = 0.239
Excpression log2(TPM+1)
Absolute Copy Number

**Supplemental Figure 2. Expression of cancer-testis genes (CTG) and somatic HR genes in tumors and normal somatic tissues.** RNA transcript expression analysis using the Gene Expression Profiling Interactive Analysis (GEPIA; http://gepia.cancerpku.cn) portal to analyze CTA/CTG and somatic HR gene expression from TCGA and GTEx datasets in normal somatic tissues and cancers [4]. The Y axis is the gene expression in $\log_2$(TPM+1) and adjusted according to the data. The X axis lists the cancers/corresponding normal tissues analyzed.

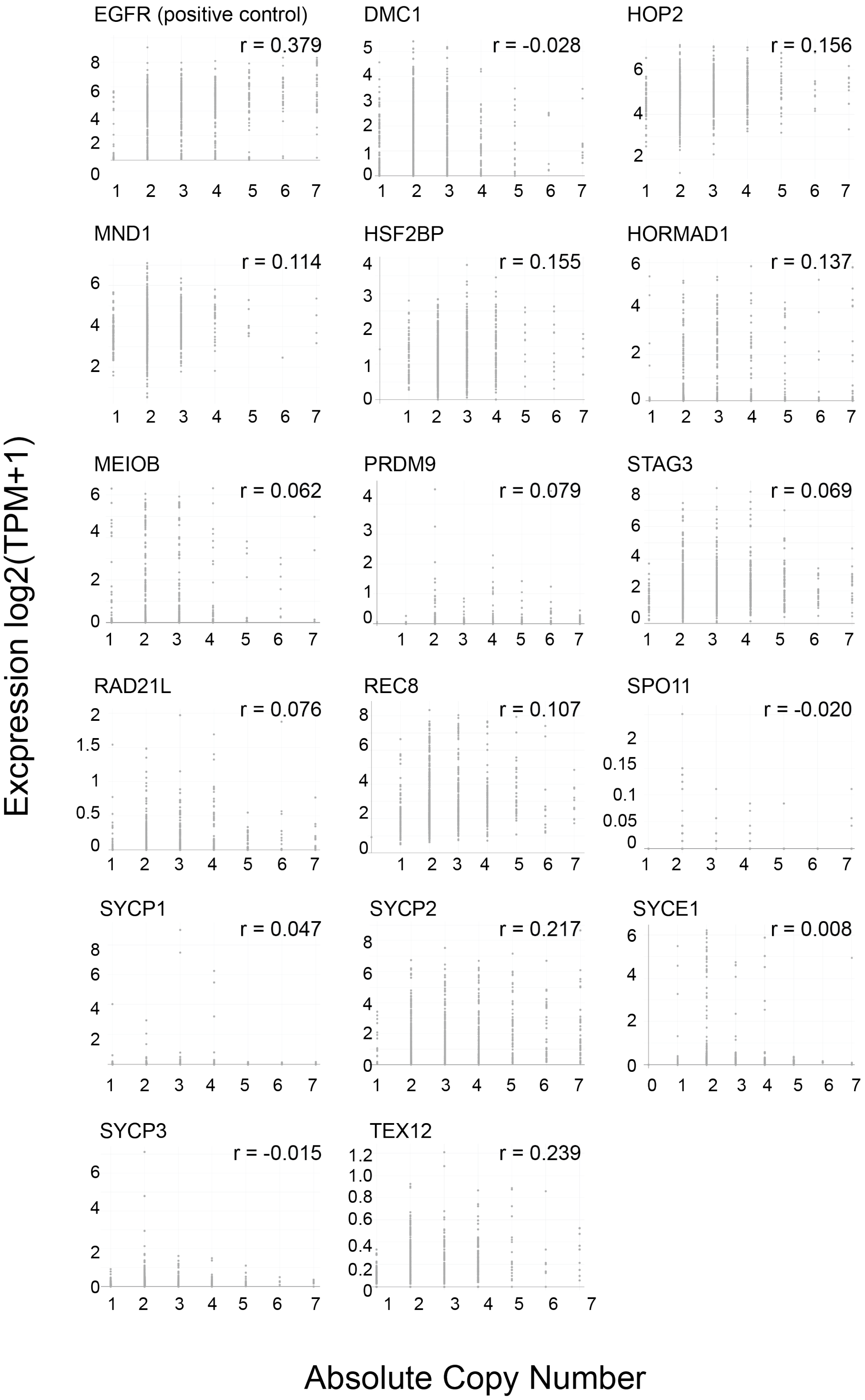
EGFR (positive control)
r = 0.379
DMC1
r = -0.028
HOP2
r = 0.156
MND1
r = 0.114
HSF2BP
r = 0.155
HORMAD1
r = 0.137
MEIOB
r = 0.062
PRDM9
r = 0.079
STAG3
r = 0.069
RAD21L
r = 0.076
REC8
r = 0.107
SPO11
r = -0.020
SYCP1
r = 0.047
SYCP2
r = 0.217
SYCE1
r = 0.008
SYCP3
r = -0.015
TEX12
r = 0.239
Excpression log2(TPM+1)
Absolute Copy Number

**Supplemental Figure 3. Correlation of gene expression with copy number.** The copy number analysis of cancer cell lines used the Dependency Map (DepMap; https://depmap.org/protal) database [1]. r is the Pearson's correlation coefficient. The Y axis is the gene expression in $\log_2$(TPM+1). The X axis is absolute copy number and adjusted according to the data. See **Supplemental Table 1** for cancer type-resolved analysis.

**Supplemental references**


1. *DepMap, Broad (2019): DepMap 19Q3 Public. figshare. Dataset doi:10.6084/m9. figshare.*
2. Dempster, J.M., Kazachkova, M., Pan, J., Kugener, G., Root, D.E., Tsherniak, A. (2019) *Extracting Biological Insights from the Project Achilles Genome-Scale CRISPR Screens in Cancer Cell Lines.* bioRxiv.
3. Shaul, Y.D., et al., *MERAV: a tool for comparing gene expression across human tissues and cell types.* Nucleic Acids Res, 2016. **44**: D560-6.
4. Tang, Z., et al., *GEPIA: a web server for cancer and normal gene expression profiling and interactive analyses.* Nucleic Acids Res, 2017. **45**: W98-W102.
